# Rethinking gene-edited crop regulation: advancing a Principle-based framework for modern biotechnology governance

**DOI:** 10.1080/21645698.2025.2576734

**Published:** 2025-11-16

**Authors:** Saarani Vengadesen, Mohammad Firdaus Bin Abdul Aziz, MichaelG.K Jones

**Affiliations:** a Faculty of Law, Universiti Malaya, Kuala Lumpur, Malaysia; b Food Futures Institute, Murdoch University, Perth, Australia, 6150

**Keywords:** Cartagena Protocol on Biosafety, Gene Editing, Genetically Modified Organisms, Living Modified Organisms, Precautionary Principle, Principle-based Approach, Process-based, Product-based

## Abstract

Gene editing (GEd) technologies are rapidly transforming agricultural biotechnology; however, their regulatory treatment remains ambiguous under international instruments such as the Cartagena Protocol on Biosafety (CPB), which was originally developed for genetically modified organisms (GMOs). This regulatory uncertainty creates challenges for product developers and regulators. This study critically examines the role of the Precautionary Principle (PP) in governing emerging genetic technologies. While the PP underpins the CPB, its interpretation, particularly in the European Union, has been criticized for creating legal barriers that have delayed the adoption of beneficial technologies. In contrast, a Principle-Based Approach (PBA) provides a more adaptive governance framework, grounded in high-level principles that enable flexibility with evolving scientific evidence. Through a review of global regulatory trends, this study identifies jurisdictional challenges and contrasts the theoretical and practical implications of the PP and PBA. It concludes with policy recommendations advocating a hybrid model integrating precaution and principle-based flexibility.

## 1. Introduction

Gene editing (GEd, also known as genome editing), provides a series of cutting-edge developments in biotechnology which promise to contribute new solutions to modern agricultural challenges such as climate change, food insecurity, and crop disease resistance. Techniques like Clustered Regularly Interspaced Short Palindromic Repeats and CRISPR-associated protein 9 (CRISPR/Cas9) enable precise genetic modifications, positioning GEd as a significant advance over Genetically Modified Organisms (GMOs). However, despite the potential of GEd, it occupies a legal gray area in international biosafety regimes, most notably the Cartagena Protocol on Biosafety (CPB), a legally binding international agreement. It was originally formulated with earlier forms of genetic modification in mind, mainly Living Modified Organisms (LMOs), legal technical term equivalent to GMOs.

According to Article 3(g) of the CPB, an LMO is defined as; “any living organism that possesses a novel combination of genetic material obtained through the use of modern biotechnology.” The objective of the CPB is to ensure an adequate level of protection in the safe transfer, handling, and use of LMOs that may adversely affect the conservation and sustainable use of biological diversity, taking into account potential risks to human health, particularly in the context of transboundary movements (CPB, 2000). Importantly, the CPB is operationalized through the Precautionary Principle (PP), which serves as its guiding legal and decision-making framework. This principle emphasizes preventive action in the face of scientific uncertainty, prioritizing safety and risk avoidance over innovation.^1^

The emergence of GEd technologies has, however, challenged this precautionary foundation.^2^ Unlike conventional GMOs, GEd allows for targeted and precise genetic modifications that can be achieved without introducing DNA from unrelated species. Consequently, certain GEd products do not meet the CPB’s definition of LMOs, as they lack a “novel combination of genetic material.” This distinction has led to divergent regulatory interpretations.^3^ For instance, the European Union applies the PP stringently in regulating GEd crops under the same framework as GMOs.^4^ In contrast, countries such as Argentina, Brazil, India, and China^6–8^ adopt a more flexible precautionary approach, exempting certain GEd products from GMO regulation when a novel combination of genetic material is absent, or when the same outcome could have been achieved by conventional plant breeding. This divergence highlights the pressing need to rethink regulatory frameworks to accommodate GEd effectively.

These differing interpretations have created a “regulatory mixture,” where countries vary in how they classify and regulate GEd products. This divergence not only complicates international trade but also underscores the need for regulatory clarity and harmonization.^9–14^ Beyond regulation, the Intellectual Property (IP) environment for GEd crops also raises concerns. The combination of patents and tight regulations raises the cost of research and smaller innovative companies will not have a chance to enter the market.^15^

The aim of this article is to present a broader perspective by linking the emergence of GEd technologies to the applicability of the PP, which has raised the question of whether the PP remains an appropriate approach for regulating GEd products,^4^ and to highlight how this connection underscores the need for an alternative approach. By examining how the PP is interpreted in legislation and identifying other regulatory hurdles, an alternative legal approach known as “Principle-Based Approach (PBA) is proposed to enable regulations to evolve and keep pace with technological innovations.

The PBA is grounded on flexible, high-level principles of safety, and risk proportionality, and offers a more adaptive and innovation-enabling framework for governing emerging technologies. This article brings a new perspective by critically evaluating the conceptual and practical implications of the PP and PBA in the context of GEd regulations. It draws on international regulatory trends and proposes a hybrid model for countries where modern biotechnology legislation is under revision, to provide better alignment of legal systems with scientific progress.

Integrating a new regulatory paradigm, however, is not straightforward. Regulators must operate within existing national and international legal frameworks. Nevertheless, the PBA can be introduced incrementally, for instance, through secondary mechanisms such as ministerial orders, regulatory guidelines, or technical annexes. This provides countries with flexibility to adapt without waiting for lengthy legislative reforms. A practical example is the Philippines, which successfully incorporated GEd into its biosafety framework through updated guidelines.^5^ Hence, we set out a forward-looking regulation framework for GEd crops, addressing the global discussion.

## 2. Literature Review

### 2.1. The Rise of Gene Editing

GEd is a set of modern biotechnology tools within the broader category of NBTs (New Breeding Techniques), also referred as genome editing. It can be distinguished from genetic engineering, which broadly relies on the random and often unpredictable insertion of isolated genes or non-native DNA elements into the plant genome. GEd, or more broadly, NBTs, introduce new traits by precisely modifying existing genes without necessarily incorporating DNA from other species.^16^ Although GEd technologies such as Zinc Finger Nucleases (ZFNs), meganucleases, and Transcription Activator-like Effector Nucleases (TALENs) have been available for some time,^17^ it was the introduction of CRISPR/Cas9 for plant gene editing in 2013 that truly revolutionized the field.^18,19^ CRISPR/Cas9 offers a simpler, more efficient, adaptable, and cost-effective tool, making gene editing highly accessible to researchers worldwide. The adoption of CRISPR/Cas9 has grown exponentially since 2013, as evidenced by the surge in scientific publications and research outputs, firmly establishing it as the preferred GEd tool for both research and commercial applications^20^ (Jones et al, 2022).

One of the most significant benefits of GEd is that it can be used to achieve precise, targeted modifications at the single base to gene level at a desired site in the genome. CRISPR/Cas9 is a major advance compared to GM techniques because it is highly specific, efficient, adaptable, and affordable, making it especially valuable for crop improvement initiatives.^21,22^ GEd is developing as a toolkit of techniques that can be used to generate targeted defined single or multiple knockouts, deletion of gene sequences, knock-ins, allele swopping, translocations, changes in gene expression, changing a single base for another, and altering epigenetic marks without changing the original DNA sequence (Jones et al., 2022^23^). It is becoming accepted as an indispensable tool for modern agricultural biotechnology.^24^

GEd can substantially accelerate crop improvement if it is recognized as distinct from GMO technology and treated as a form of conventional breeding, as long as no novel traits are introduced. For instance, the approval of drought-tolerant soybeans and oil-enriched flax by the U.S. Department of Agriculture (USDA), or the high GABA Sicilian Rouge tomatoes for sale in Japan, demonstrate how GEd crops can meet regulatory requirements while not subjected to extensive GMO review, which is costly and length.^25^ While such advances hold great promise to support sustainable agriculture and global food security, they also raise issues for existing national regulatory frameworks.^26,27^ For example, GEd can be used to make changes in a genetic sequence that are indistinguishable from those which can be achieved using conventional plant breeding or occur from accepted natural or induced mutations.

The advent of GEd technologies initiated a debate amongst regulators, scientists, and stakeholders, focusing on whether organisms developed through GEd should be governed under existing GMO regulations or required a different regulatory approach. Although the CPB was initially established to oversee products of modern biotechnology, its relevance has resurfaced in light of GEd advancements. As a contribution to this ongoing discourse, we have examined some legal uncertainties that relate to the regulation of GEd crops, through reviewing definitional ambiguities and the diverse regulatory approaches adopted worldwide, and provide some recommendations for an adaptive regulatory framework.

### 2.2. Regulatory Lag: The Pacing Problem in Governing Gene Editing

Building on the earlier discussion of the rapid increase in GEd technologies such as CRISPR/Cas9, and the related regulatory uncertainties, this section conceptualizes the gap between technological innovation and regulatory adaptation. Ivanov et al.^28^ discussed the concept of regulation theoretically, noting that scientific literature related to gene technology often overlooks the term “regulation.” Regulatory hurdle or legal uncertainty has been identified as a stumbling block to the adoption of GEd technology in various literatures,^26,27,29^ Lassoued et al., 2018.^12^ With the rapid pace of technological development, a key challenge is whether legislation, regulation, and judicial mechanisms can keep up with scientific advances. Moses^10^ observed that legal systems often struggle to adapt to emerging technologies. This mismatch, commonly called the “pacing problem,” describes the failure of law and policy to evolve at a rate that matches technological progress.^11^ The pacing problem can derail emerging technologies by discouraging innovation, delaying beneficial applications, and impeding potential societal and public benefits.

The regulatory uncertainty related to GEd exemplifies this pacing problem. As GEd technologies are developing rapidly, regulatory frameworks lag, leading to governance gaps which can stifle innovation in agricultural biotechnology. Although GEd falls under the broader definition of modern biotechnology, many regulatory authorities continue to debate whether or how GMO definitions relate to GEd crops.^30^ The widely used Site-Directed Nuclease (SDN) classification system was introduced to guide regulatory decisions, but differing precautionary approaches across jurisdictions have led to a “regulatory mixture” that complicates international trade and innovation.^12^ Nevertheless, while regulation is often cited as a bottleneck, it also has the potential to be structured in a way that aligns technological developments and applications with societal expectations (Lassoued et al., 2021).^31^

### 2.3. Evolving Regional Regulatory Models

The evolving global regulatory landscape of GEd shows a significant heterogeneity from strict process-based systems to flexible product-based approaches,^32^ for example between regions such the European Union, Africa, Asia, and Latin America.^33,34^ Most countries regulate GEd crops based on the Site Direct Nuclease (SDN) technique used, namely SDN-1, SDN-2, and SDN-3. SDN-1 is site-directed mutagenesis; SDN-2 includes templated editing, and SDN-3 is site-directed gene insertion.^40^ With countries taking differing positions on whether to classify SDN-1, SDN-2, and SDN-3 as GMO, non-GMO or establish a new guideline, it has resulted in a fragmented global regulatory landscape^13^ Sprink et al., 2022.^20,41^ Most countries exempt SDN-1 from GMO regulations because it generates site-specific random mutations that can be indistinguishable from natural mutations or changes which occur in conventional plant breeding. With regard to SDN-2, the definition differs from country to country (Jones et al., 2022). For an example, in the Philippines, GEd with insertions of 19 or lesser base pairs is considered SDN-2 and exempted from GMO regulations.^42^ SDN-3 is usually subjected to GMO regulations because it involves a sequence donor, usually double-stranded DNA carrying a gene or an even longer genetic element which can include novel genetic material.^9^

Tachikawa and Matsuo (2023) classified GEd in four regulatory models: (1) treating GEd products as GMOs under existing GMO regulations; (2) applying simplified GMO regulations with reduced requirements; (3) exempting certain GEd products but requiring confirmation by the regulatory authority; and (4) granting exemptions without requiring regulatory confirmation. The United States and Canada have not adopted the SDN terminology in their regulatory frameworks. Instead, both countries follow a product-based approach, focusing on the traits and risks of the final plant product rather than the GEd process or classification by SDN type.

The evolving regulatory landscape reveals that with the adoption of SDN terminology, countries having their own definitions for SDN-2, and subjecting SDN-3 as GMOs increases the complexity in regulating GEd (Jones et al., 2022). Inconsistent regulatory approaches across regions create trade barriers for GEd products, leading to higher compliance costs, slower approvals, and constrained market entry.^20^ Developers are therefore required to navigate diverse regulatory frameworks, requiring adaptation to local rules and often additional testing, documentation, and procedures that vary by country.^32^

Moreover, divergent national rules on what constitutes a GMO versus a non-GMO add further uncertainty over patent scope and enforcement, especially for innovations developed using SDNs. For instance, a GEd crop exempted from GMO regulations in one country may face strict oversight in another, complicating freedom-to-operate assessments,^30^ cross-border licensing, and technology transfer. Breeding companies may need to obtain multiple licenses, which increases costs. GEd faces patenting issues, as courts and patent office struggle to define the boundaries of patent eligibility. The patentability of GEd became debatable when the novelty of the CRISPR system was questioned by the US Supreme Court in 2013, if it is an invention or merely nature discovered.^8^

### 2.4. Benefits of Regulatory Exemptions for Gene Editing

There are strong arguments for advocating the exemption of most GEd applications from regulation as GMOs, both because of potential economic benefits and the need for investment certainty.^2,7,43,44^ Countries that have exempted SDN −1 from being GMOs are China, Brazil, Japan, Australia, Thailand and India.^2,45^ estimated that regulating a GEd crop as a GMO could cost in the order of USD 24.5 million and take up to 14 years to reach the market. In contrast, if GEd crops are regulated as conventionally bred crops, costs drop to about USD 10.5 million, and the timeline can shorten to less than 5 years. Thus, regulatory efficiency fosters broader market participation and accelerates innovation.

Ramsay et al.^46^ argued that regulation should be proportionate to the risk-benefit profiles of NBTs, as overly burdensome processes exclude most smaller enterprises and nonprofit developers. Most of the Latin American countries have adopted more innovation-friendly regulatory frameworks for GEd crops. Argentina, Brazil, Chile, Colombia and Paraguay adopts a case-by case assessment of products obtained through GEd. When no novel genetic material is present and the genetic change could have occurred through conventional breeding, such products are increasingly being exempted from GMO regulations.^32,35,47^ This approach streamlines market entry, offers greater regulatory certainty for developers, and encourages investment by minimizing costly and time-consuming approval processes.^32^ Evidence from Argentina, the first country to implement specific guidelines for GEd products, shows the tangible benefits from reduced regulatory barriers. Argentina’s Prior Consultation Instances (PCIs) serve as a leading example of how this model can effectively facilitate agricultural innovation.^36^

The high cost of GMO commercialization and lengthy approval times have clearly confined innovation to a narrow range of traits and developers.^37^ Kalaitzandonakes et al.^44^ estimated the average regulatory compliance cost for a single GM trait in one market to be USD 7.8 million, increasing to USD 55 million when targeting multiple markets. The regulatory process for new GM crops or traits usually involves extensive assessment of possible unintended effects, extending market entry timelines substantially. Conversely, GEd applications could take less than 5 years and cost the same or slightly more than conventionally bred varieties. More rapid deployment supports the strategic emphasis on enhancing food systems through targeted innovation and wider participation.

## 3. Theoretical Framework: Precautionary Vs. Principle-Based Regulation

### 3.1. The Precautionary Principle

The basic idea of the PP is that development of a technology must be regulated if there is concern of possible impacts to human health and the environment. However, previous experience in regulating GMOs indicates that it is important to understand the basic terminologies related to PP. Caradus,^4^ Ishii,^48^ Sinebo and Maredia,^49^ discussed how misinterpretation of terminologies has led to stringent implementation of the CPB which was formulated based on Principle 15 of the 1992 Rio Declaration on Environment and Development. In the specific statement the terminology “precautionary approach” was used.

Principle 15 of the 1992 Rio Declaration on Environment and Development, the:

Precautionary Approach states that where there are threats of serious or irreversible damage, lack of full scientific certainty shall not be used as a reason for postponing cost-effective measures to prevent environment degradation.^50^

Instead of the term precautionary approach, the European Union adopted the “Precautionary Principle” in their legislation.

The European Commission Communication on the PP states that:

The precautionary principle applies where scientific evidence is insufficient, inconclusive or uncertain and preliminary scientific evaluation indicates that there are reasonable grounds for concern that the potentially dangerous effects on the environment, human, animal or plant health may be inconsistent with the high level of protection chosen by the EU.^51^

Although, both the terms sound similar, they have distinct meanings as defined in Table below. The Precautionary Approach refers to how a particular country, organization, or individual decides to apply the PP when assessing potential risks.^4^ This accounts for varying regulatory approaches to GMOs as countries can determine their own application and emphasis of the PP. Table 1 shows the terminology related to precaution.

**Table 1.** Terminology related to precaution.^52^

Precaution	Webster’s dictionary defines precaution “as a measure taken beforehand against possible danger.”
Precautionary Approach	A regulatory approach, such as that applied by the United States, that seeks to err on the side of safety by applying precaution informally and implicitly in regulatory decisions.
Precautionary Principle	A legal requirement, such as that enacted by the EU, that mandates the formal and explicit application of precaution in regulatory decisions.

There is criticism of the concept of the PP itself. Peterson^38^ argued strongly that the PP should not be used as a basis of decision making, as previously noted by Myers^39^ that the PP is vague and has conflicting definitions.

The 1998, Wingspread Statement on the PP defined the principle as:

When an activity raises threats of harm to human health or the environment, precautionary measures should be taken even if some cause-and-effect relationships are not fully established scientifically^56^.

The Cartagena Protocol on Biosafety says:

Lack of scientific certainty due to insufficient relevant scientific information … shall not prevent the Party of import, in order to avoid or minimise such potential adverse effects, from taking a decision, as appropriate, with regard to the import of the LMO in question (CPB, 2000, Art. 10(6)).

The definitions above show that there is not a universal, clear-cut definition of the PP, thus the conditions or frames for its implementation are not clear, as recognized in Council for Agricultural Science and Technology (CAST),^52^ that the PP is ambiguous and lacks definition. Thus, different stakeholders may interpret precaution differently.

Various studies have been undertaken that aimed to identify the key features of the PP (Table 2). Understanding the rationale behind the PP is crucial because it is often claimed that the principle is paralyzing and unscientific and promotes a culture of irrational fear. To give weight to the PP, two main conditions are required; first the risk threat must be considered high, second the uncertainties involved must be scientific in nature such as molecular and genetic assessment, allergenicity test and environmental risk assessment and not stemming from political concerns.^57^

**Table 2.** Core elements of the precautionary Principle according to various Sources.^57^

Authors	Core elements of the PP
Bourguignon,^59^	Risk governance (risk, assessment, management and communication) Science-policy interfaces The link between precaution and innovation.
COMM (2000)	Existence of risk perceived as a threat of harm Presence of scientific uncertainty regarding potential effects Corresponding action by stakeholders.
Kriebel et al.,^60^	Taking preventive action in the face of uncertainty Shifting the burden of proof to the proponents of an activity. Exploring a wide range of alternatives to possibly harmful actions. Increasing public participation in decision-making.
Sandin et al.,^61^ Rechnitzer^62^	Threat Uncertainties Actions/Commands

Based on Table 2, the three main features of the PP are risk assessment, scientific uncertainties, and participation of all key stakeholders. It is essential to understand the key features of the PP because the elements are reflected in the CPB as discussed below.

### 3.2. Reflection of the Precautionary Principle in the CPB

Examining how the PP is reflected in the CPB is necessary because the extensive regulatory assessment of GM plant technologies has contributed to non-harmonious and asynchronous approvals delayed or prevented the commercialization of many GM crop events, increased costs, and complicated market access.^43^ Without greater alignment or adaptive regulatory frameworks, similar challenges could hinder delivery of the potential benefits of GEd for global agricultural production, consumers and food security. The PP is reflected in several provisions of the CPB, which integrate the principles outlined in Principle 15 of the Rio Declaration on Environment and Development. Key elements include:

- **The Preamble**: Reaffirms the precautionary approach, referencing Principle 15 of the Rio Declaration, emphasizing the need to take precautionary measures in the face of scientific uncertainty regarding potential environmental and health risks.
- **Article 1**: States that the objective of the Protocol is to act “in accordance with the precautionary approach,” ensuring that actions are taken to protect biodiversity and human health, even when scientific knowledge is incomplete or uncertain.
- **Articles 10.6 and 11.8**: These articles clarify that lack of scientific certainty should not prevent decisions on the import of living modified organisms (LMOs). If there is uncertainty regarding potential adverse effects, countries can still make decisions to prevent or minimize risks, reinforcing the role of precaution in managing biotechnology.
- **Annex III on Risk Assessment**: Stresses that lack of scientific knowledge or scientific consensus should not be taken as evidence of no risk or an acceptable risk. This aligns with the PP, asserting that decisions should err on the side of caution when the potential risks of an LMO are not fully understood.

These provisions in the CPB ensure that the PP is applied consistently in regulating modern biotechnology, as in the Preamble and Article 1, the term precautionary approach has been used, Article 10.6, 10.8, addresses scientific uncertainty, and Annex III focuses on risk assessment.

### 3.3. The Precautionary Principle: Linking LMO Definitions, Process-Based Triggers and Gene Editing Risk Assessment

The PP plays a foundational role in shaping the definition of LMOs under the CPB and implements adopting a process-based regulatory approach. The CPB explicitly incorporated the PP in its preamble, Article 10 and 11 as discussed above. This principle underpins the process-based definitions of LMOs which focus on whether an organism was developed using modern biotechnology, a precautionary trigger that prioritizes how an organism was made rather than what trait it expresses. This approach was justified at the time by the scientific uncertainty surrounding the potential risks of recombinant DNA techniques, which introduced exogenous genetic material and created novel combinations not previously observed in nature.^63^ However, the emergence of GEd, especially those involving non-transgenic edits or modifications using only endogenous DNA, challenges the CPB’s original regulatory framework.

As the scientific uncertainty that originally justified the application of the PP to GMOs is considerably reduced in the case of precise and well-characterized GEd applications,^40^ NBTs blur the line between conventional breeding and “genetic modification,” especially since they can generate organisms that are indistinguishable from those produced through natural or traditional methods.^26^ The scope of LMO in CPB, primarily covers organisms containing a novel combination of genetic material obtained through recombinant DNA methods, however the size of the nucleic acid insert is undefined in CPB.^40^ In contrast, the end -products of SDN-1 methods do not contain inserted nucleic acid, and so do not meet the definition of an LMO in the CPB.

Thus, the concept of scientific uncertainty and process-based approach no longer adequately capture GEd applications. Sandin^64^ has cautioned that PP application has many ill-defined factors, such as “lack of scientific uncertainty” and “unacceptable harm,” which makes it susceptible to manipulation. For instance, the CPB’s formulation of the PP specifies that “lack of scientific certainty … shall not prevent” regulatory decisions (Articles 10 and 11). In practice, this often been interpreted in reverse, where uncertainty is treated as a reason for inaction rather than a basis for precautionary decision-making. Thus, applying the PP in this evolving scientific and regulatory context thus necessitates a reexamination of how regulatory thresholds are defined.

### 3.4. Hurdles in Regulating GEd Crops

Based on the literature, four main legal hurdles have been identified for regulating GEd crops:

- Applicability of the PP
- Legal definitions and the scope of the definition of a GMO
- Process vs product approach
- Risk assessment complexity

#### 3.4.1. Applicability of the Precautionary Principle

With the emergence of NBTs, the application of the PP in the context of scientific uncertainty has been criticized widely.^52,53,54,65^ Over the past 30 years, the overly cautious regulatory stance has significantly constrained the commercialization of GM crops in the European Union and elsewhere.^54^ Caradus^4^ noted that many countries have merged precaution with prevention, while Sinebo and Maredia^49^ argued that, although some countries interpret precaution as a basis for scientific risk assessment, many developing nations have enacted overly prohibitive and unworkable legislation. Legislation is considered overly prohibitive when its requirements impose cost, delays, or restrictions disproportionate to the actual risks, thereby limiting innovation.^66^ It is discussed in Evolving Regional Regulatory Models on how regulatory differences is affecting global trade and impose delays.

Ludlow et al.^67^ traced the emergence of PP critiques to the mid-1990s, when regulatory approaches to GM crop approvals separated into two main groups. One group, comprising countries such as Argentina, Brazil, Canada, and Japan, while the other, primarily the European Union and several Asian countries, pursued stricter precaution-based regimes. While both groups use scientific risk assessment and variety approval processes, they differ significantly in how heavily they rely on the PP.

Lassoued et al.^2^ argued that the rise of GEd technologies exposes the limitations of precaution-based governance. Critics contend that the PP is often applied as a regulatory endpoint rather than as a structured, evidence-based tool to ensure safety. In practice, it has been used to justify regulatory decisions without transparent reasoning, leading to inconsistencies, reduced predictability, and limited accountability.^68,53^

Aven^57^ similarly cautioned against an overly simplistic interpretation of the PP as merely “better safe than sorry,” warning that such generalizations blur the boundaries of science-informed regulation. Further critiques view the PP as a political rather than scientific tool, arguing that it has been used to block the adoption of beneficial technologies or as a non-tariff trade barrier.^58^ In the case of the European Union, the GMO regulatory system is often described as politically driven,^55^ with lawmakers and courts invoking the PP to justify restrictions.^4^ While there is broad scientific evidence supporting the safety of many GMOs^69,70^ the issue has long been controversial, including among scientific experts, and regulation in democratic societies is expected to be politically shaped, though ideally not captured by partial interests.^99,100,101^

#### 3.4.2. The Definition Puzzle: Interpreting GMOs, LMOs, and Gene Editing

The emergence of NBTs presents fresh challenges for regulators tasked with interpreting existing definitions of GMOs or LMOs.^26,40,46^ Many current biosafety laws do not adequately account for the specific features of NBTs. A central issue is the definition of GMO that has undergone an important shift. Initially, GMOs were broadly described as organisms “modified by recombinant DNA (rDNA) techniques.” Over time, however, regulatory and public discourse has increasingly equated GMOs with organisms that contain *exogenous* DNA.

This evolution in terminology has significant implications for GEd, particularly those developed through SDN-1 approaches, which may not contain exogenous DNA in the final product, thereby challenging traditional GMO definitions and their regulatory applicability.^8,47^ Mackenzie et al.^71^ and Schmidt et al.^13^ have pointed out that the CPB’s definitions, particularly of terms like “novel,” “modern biotechnology,” and “genetic material,” lack clarity, contributing to legal uncertainty. As a result, some Parties apply case-by-case assessments, while others enforce blanket regulations, often creating inconsistencies in how GEd crops are governed.^26^

In response to such definitional and legal ambiguities, the European Union formally introduced the term *New Genomic Techniques* (NGTs) in its 2023, legislative proposal to establish specific rules for plants developed using GEd methods. The European Union is the only region where the term NGT has gained official recognition in legislative discourse. This term reflects the EU’s acknowledgment of the need to revise existing regulations in the light of biotechnological advances that do not align with traditional GMO definitions.^72^ The proposed *Regulation on Plants Obtained by Certain New Genomic Techniques* aims to modernize the European Union’s long-criticized process-based regulatory system by creating a separate category for NGTs, defined as genome-targeting techniques developed after 2001.^73^

NGT plants are defined as plants that have been modified by targeted mutagenesis or cisgenesis, targeted mutagenesis being defined as mutagenesis techniques resulting in modification(s) of the DNA sequence at precise locations, and cisgenesis as genetic modification techniques resulting in the insertion of genetic material already present in the plant’s gene pool. In the newly established procedure it must be verified if the plant is derived by NGT and it has further two classifications:^74^

- **NGT Category 1**: Plants that are exempted from authorization for deliberate release or bringing to the market, risk assessment for human health and environment and requirements of co-existence with non-GM agriculture. Monitoring and labeling as GMO are curtailed.
- **NGT Category 2**: Remains subjected to the current regime for GMOs, but with relaxed risk assessment, shortened timelines, reduced monitoring and certain incentives toward steering genetic modifications to more sustainability.

This classification system represents the EU’s attempt to balance innovation with public concern over GMOs. By contrast, most other jurisdictions have not adopted the NGT terminology formally, although they have updated regulatory practices to accommodate GEd products. Table 3 shows how the concept is applied in different regulatory frameworks. This divergence highlights the global regulatory fragmentation relating to GEd.

**Table 3.** Terms and regulatory framework for biotechnology regulation across selected countries.

Country	Regulatory Framework	Term
United Kingdom	UK Genetic Technology (Precision Breeding) Act 2023	Precision Breeding
European Union	EU Directive 2001/18/EC	Genetically Modified Organisms (GMO), New Genomic Techniques (NGTs)
United States of America	United States Coordinated Framework for Regulation of Biotechnology	Genetic Engineering
Canada	Canada Seeds Regulations	Plant with Novel Traits (PNTs)
Malaysia	Malaysia Biosafety Act 2007	Living Modified Organisms

In June 2025, Food Standards Australia New Zealand (FSANZ) announced a major update to how genetically modified food is defined under the Australia-New Zealand Food Standards Code. As part of Proposal P1055, the outdated process-based definition of *“food produced using gene technology”* has been replaced with a clearer, outcome-based definition of *“genetically modified food,”* which also includes food derived using SDN-1 and 2. This new definition focuses on the presence of *novel DNA*, genetic material that is new or altered in a way that does not occur naturally or through conventional breeding. Changes resulting from natural processes or traditional breeding will not be captured under the GM classification, improving regulatory clarity and keeping pace with advances in gene technologies.^75^

#### 3.4.3. Process or Product? Regulatory Paradigms for Gene-Edited Crops

The application of process-based vs product-based regulatory approaches has been the subject of considerable debate after the emergence of GEd.^4,6,63,76^ Process-based regulatory approaches focus on the technology or process applied to develop the final product, which is typically triggered by the use of recombinant DNA technology rather than by the features or risk of the resulting organism. In contrast, a product-based regulatory approach assesses organism based on the characteristics of the final product if it has a novel trait, regardless of the method used to generate them and focuses solely on whether the final product presents risks to humans or the environment. Canada’s regulatory model for “plants with novel traits” exemplifies this approach.^6^

GEd has fueled debate on whether a product-based approach would provide a more scientifically sound regulatory framework because products derived from SDN-1 techniques can be indistinguishable from those produced through conventional breeding, as the genetic changes introduced could also occur naturally or through traditional methods. Although the techniques used are different, the outcomes are similar raising the question of why regulation should focus on the process rather than the final product.

GEd does not pose inherently greater risks than conventional breeding and recommended a shift toward product-based regulation rather than focusing on the process used.^77^ Moreover, similar genetic changes can arise naturally through mutations, recombination, or horizontal gene transfer, further challenging the rationale for process-based oversight.^32^ From a biosafety perspective, the risks of some GEd products should therefore be assessed in line with those of naturally occurring genetic variation or conventionally bred plants.^78^ Despite the arguments, most countries still follow a process-based approach and few countries have adopted product-based approach as shown in Table 4.

**Table 4.** Comparison of process-based vs product-based regulation.

Approach	Focus	LMO Compatibility	Example countries
Process-based	The method used to modify the organism (recombinant DNA, CRISPR)	Fully aligned with the CPB’s LMO definition	European Union, Malaysia, India, Australia, China
Product -based	The final traits or characteristics of the organism, regardless of how it was made	Not inherently compatible - some GEd organisms might escape regulations.	USA, Canada (for novelty), Argentina in some cases

Product-based regulation supports a more proportionate, risk-based framework better suited to accommodating innovation^79^ and fosters regulatory coherence and fairness.^63^ The^72^ study acknowledged that the current process-based GMO legislation is no longer fit for purpose, particularly in the context of GEd. The enforcement of process-based regulations becomes technically unworkable when it is impossible to determine whether a product was developed using a specific technique. For instance, if a mutation created through CRISPR/Cas cannot be distinguished from one occurring via conventional mutagenesis, regulators face significant challenges in ensuring compliance and detecting unapproved GMOs in seeds. This undermines the regulatory goals of traceability and safety assurance.^6^ While some scholars argue against framing the debate as a strict process vs. product dichotomy and instead advocate for integrative regulatory approaches.^80^ McHughen^76^ pointed out a practical flaw in the process-triggered model: as scientific methods continue to evolve, regulators must continually assess whether each new technique falls within the scope of existing laws, often requiring costly and time-consuming legislative amendments.

#### 3.4.4. Risk Assessment Complexity

The next regulatory hurdle is the complexity of risk assessment. The current risk assessment approach is not designed to detect unintended outcomes from GEds. To address this, untargeted metabolomics could be incorporated as part of a routine protocol assessing future biotech crops.^81^ Risk assessment for GEd plants will have to consider a broader spectrum of new genetic combinations and traits compared to the limited number of traits commercialized by first-generation genetic engineering resulting in GM crops. A one-size-fits-all regulation is not appropriate to deal with a wide variety alterations and risk profiles. GEd varieties may require more detailed risk assessments for complex changes involving many genes or possible off-target changes.

Risk assessments were developed primarily for transgenic organisms and may not be well suited to evaluating the subtle, precise changes made by GEds.^2^ In particular, small gene edits that do not introduce foreign genes can be difficult to detect using conventional methods of molecular characterization.^13^ In addition, many countries still apply risk assessment procedures designed for previous GMO technologies, and the absence of harmonized, updated guidelines for GEds contributes to regulatory uncertainty and potential gaps in identifying risks.^83^

Conventional mutagenic treatments result in many more double-stranded breaks in DNA than may occasionally result from CRISPR processes. Crop varieties selected after irradiation or chemical mutagenesis do not require any form of risk assessment. Products derived from SDN-1 and in selected case of SDN-2 also often produce alterations indistinguishable from those occurring through natural mutations or conventional breeding, but there is still a desire by some to determine off-target edits in GEd products, and current regulatory frameworks do not require techniques such as whole-genome sequencing to determine if off-target edits have occurred, or whether that level of assessment in actually needed given the natural sequence variation present in food crops, as clearly shown from pangenome sequencing.^26^

Finally, in jurisdictions with product-based regulations, unintended effects might not be studied if the final product appears like conventionally bred crops, including when modern biotechnology was used to develop it.^82^ However, countries like India and China have reduced their risk assessment complexity (Rios Fernandez et al., 2025). Since, 2022, China has implemented regulations that shorten the approval times for products derived from NBTs to 1–2 years. This framework prioritizes food safety and environmental impact assessments. Meanwhile, India has adopted a flexible approach by exempting SDN-1 and 2 from GMO regulation provided no transgene is present.^45^

### 3.5. The Principle-Based Approach: A Flexible and Adaptive Alternative

PBA offers a more adaptive and responsive framework for regulating emerging technologies. Originally proposed by Carter and Marchant^84^ for the United States context, PBA moves away from rigid rules and emphasizes guiding principles such as safety, proportionality, transparency, and ethical responsibility to assess new technologies within their specific contexts. However, because interpretations of these principles can vary significantly between countries, there is a risk of regulatory fragmentation. To avoid this, guiding principles can be formulated jointly at regional levels. For example, through ASEAN, the European Union, or Asia-Pacific Economic Cooperation (APEC), to promote greater consistency and harmonization in regulatory practices.

For an example, Australian and New Zealand, has a joint food regulation system managed by FSANZ with a unified safety criteria.^75^ Unlike standards that prescribe specific requirements, the PBA relies on broad principles rather than detailed, prescriptive rules,^85^ enabling greater flexibility and timely regulatory responses to technological advances without the constant need to draft new legislation, making PBA a potentially powerful mechanism for both innovators and regulators to be agile in the face of rapid innovations.^84^

Although the PBA has not been formally adopted in biotechnology governance, its influence is growing in fields such as artificial intelligence and financial services. For example, Arner et al.^86^ conceptualized a PBA for governing BigFinTechs, and Weidener and Fischer^87^ advocated its use in teaching AI ethics in medical education. In biotechnology, elements of the PBA are present in countries like Argentina and Brazil, where GEd products are regulated based on the characteristics of the final product rather than the process used to develop them. While the PBA promotes regulatory flexibility and innovation, it also poses challenges.

Broad principles may result in inconsistent interpretation or regulatory discretion, potentially undermining legal certainty and public trust. However, these concerns can be mitigated through robust institutional frameworks and stakeholder engagement. Marchant et al.^88^ proposed that PBA could serve as a suitable interim regulatory framework for emerging technologies. Such an approach can be implemented relatively quickly and provide preliminary oversight while a more traditional, rule-based system is developed. For instance, the European Union introduced a code of practice for nanotechnology researchers grounded in general principles, acting as a gap-filling measure until more detailed regulatory structures were established.^65,89^

Table 5 provides a comparative overview of the key characteristics of the PP and PBA.

**Table 5.** Comparison of the pp and the PBA.

Aspect	Precautionary Principle (PP)	Principle-Based Approach (PBA)
Basis	Risk aversion under uncertainty	Guiding principles and values
Focus	Process (how it’s made)	Product (what it is)
Regulatory Style	Rigid, rule-based	Flexible, context-driven
Innovation Impact	Often stifling	Enabling, if well-managed
Example	EU GMO Regulation	Canada’s product-based model

## 4. Discussion: Applying Regulatory Principles in Practice: Why Principle-Based Regulation May Be a Better Fit

A PBA to regulating GEd provides an opportunity to address many of the challenges that current definition-driven frameworks face. Earlier regulation often hinges on whether an organism meets specific legal definitions, such as GMO or LMO, terms that can be ambiguous when applied to GEd crops. This results in legal uncertainty, inconsistent decision-making across jurisdictions, and regulatory misalignment with scientific understanding as discussed extensively in the earlier part of this paper. In contrast, a PBA focuses on core regulatory values such as scientific evidence, risk proportionality, and transparency, rather than on how a product was made.

While it is true that certain contextual information about the process may help interpret scientific evidence, a key principle of the PBA is that risk assessment should be based primarily on the characteristics and intended use of the final product, not the method used to create it. Considering the process risks reintroducing process-based triggers, which the PBA seeks to avoid. Scientific evidence under PBA is evaluated for its relevance to product traits and potential hazards, regardless of how those traits were achieved. Therefore, while process data can inform understanding, it should not determine regulatory classification or decision-making. This view aligns well with decades of empirical evidence demonstrating that risk depends on the function and expression of novel traits, not the method of their introduction.^6,90,91^

Canada provides a leading example of this approach through its *product-based* regulatory system. Rather than regulating organisms based on whether they were produced using a particular technique, Canada assesses Plants with Novel Traits (PNTs), whether those traits arise through conventional breeding, mutagenesis, or modern biotechnology. This principle-driven model ensures that oversight is applied where it is scientifically warranted, not simply because a specific method was used. It allows for greater flexibility in addressing NBTs, avoids unnecessary regulatory burdens for low-risk products, and reduces barriers to innovation.^26^

It is necessary to adopt a PBA because, beyond regulatory clarity, GEd faces additional challenges that will influence technology adoption. Two significant challenges are IP and patents^92^ over the main GEd technologies, which allow public R&D but require one or more licenses for commercial applications, and public awareness. Patents on core tools, such as CRISPR-Cas9, are likely to reduce access to GEd technologies for small breeders and public research institutions, concentrating commercial applications to a few well-resourced companies or other organizations. As for GMOs, these dynamic risks may increase technology gaps between developed and developing countries, even when regulatory frameworks are supportive. Similarly, limited public awareness and understanding of GEd present a barrier to consumer acceptance.^93^ Addressing these additional issues requires a science-based and transparent approach that promotes stakeholder inclusivity, for which the PBA offers a better regulatory fit.

In the ISAAA Brief No.56, criteria for an ideal safety regulation to reduce the regulatory burden and facilitate innovation was being proposed as shown in Table 6.^94^ The criteria align well with the criteria of PBA, which reflects that a PBA will be a better fit.

**Table 6.** Intersection of proposed criteria with PBA.

Proposed criteria	How it aligns with PBA
Fit for purpose	Allow jurisdictions to adopt implementation while respecting common high-level policy, safety and transparency principles.
Science-based regulation	Evidence and Transparency Principle: Used best available science and peer-reviewed data; enhances trust and consistency internationally.
Risk proportionate regulation (Stringency based on actual risk level)	Discourages loophole behaviour and a checklist-style approach.
Product-based focus	Outcome-oriented: Focus on final products instead of the process.
International harmonization	Encourages convergence based on shared principles rather than identical regulatory texts.

Adopting a PBA can promote international harmonization as it follows a product-based approach and common guiding principles among regions. By shifting the focus from the process-based approach which varies between countries, to shared principles of risk assessment, countries can reduce regulatory divergence. This regulatory approach encourages more effective regulation by discouraging “loophole behaviour and checklist-style approaches.”^95^ This criterion of a PBA is well aligned with Whelan and Lema,^7^ who argued that new regulations should not be based on a closed list or description of technologies, rather they should be framed to be flexible and applicable to existing and future technologies.

### Toward a Hybrid Model

As discarding the PP altogether will not be practical because GEd still falls under the umbrella of modern biotechnology and needs to comply with the CPB, a hybrid regulatory model may offer the most pragmatic path forward. Such a model would retain the PP’s core idea of protecting humans and the environment, and establish guiding principles consistent with CPB. Table 7 shows how PBA can be applied by formulating guiding principles in the context of GEd.

**Table 7.** Guiding principles to regulate GEd.

Guiding Principle	Purpose
Science-based risk assessment	Decisions rely on evidence which evaluates molecular genetic assessment, allergenicity, toxicity and nutritional assessments, environmental risk assessment based on empirical and peer-reviewed methods.
Product-based approach	Evaluate based on the final product and not the process such as GMO or GEd.
Enables Risk-Proportionate Regulation	Develop clear criteria to direct whether a product requires full risk assessment or a simplified pathway. Example Case A: SDN-1 edit (small deletion in a gene) If an unintended nearby mutation occurs but does not produce a harmful trait, PBA considers it low risk, like a natural mutation. Case B: Gene editing creates unexpected protein expression PBA requires further safety testing before approval, but only for that specific risk, avoiding unnecessary regulation of unrelated edits.
Transparency	• Establish clear timelines for decision-making to prevent bottlenecks. • Set up periodic review cycles so the regulatory system evolves as new evidence emerges.

This guiding principles can be applied to regulate GMO as well but with particular emphasis on assessing the presence and characteristics of novel genetic material. It might raise a question of how PBA can handle unintended genetic changes. They are assessed based on their relevance to risk, rather than being automatically treated as GMO. This is similar to how countries in Africa, Burkino Faso, Ethiopia, Kenya and Nigeria are moving toward an adaptive regulatory framework. These African countries are regulating GEd based on the principles of case-by-case review and risk proportionality. Kenya and Nigeria have developed guidelines that distinguish between conventional, intermediate, and transgenic products, applying different levels of regulation depending on the nature of genetic modification.^96^

In the context of GEd regulation, PBA focuses on whether an unintended alteration results in a novel trait or function that could impact safety, such as producing a toxin, allergen, or posing an environmental threat. If no risk-relevant trait is expressed, additional regulation is unnecessary. This is consistent with conventional breeding methods, such as mutagenesis or cross-breeding, which also create many unintended mutations, most of which are harmless. Regulators rely on scientific evidence, including molecular characterization, compositional analysis, and phenotype testing, to determine whether any unintended effects pose a biological or ecological concern. Rios Fernandez et al., (2025) proposed that molecular characterization requirements should be limited to the species level when the edited trait falls within the range of naturally present of induced variation. Requiring varietal-level analyses in such instances imposes an unnecessary technical burden and risks regulatory disproportionality.

## 5. Conclusion and Policy Recommendations

GEd technologies are reshaping the trajectory of agricultural innovation. However, existing regulatory regimes, largely based on definitions and assumptions developed for earlier forms of genetic modification, are increasingly inadequate to address the complexities and nuances of these emerging technologies. By introducing the PBA, this article opens a new approach to exempt GEd product from GMO regulation unless it triggers a novel combination of genetic material. While scholars have previously advanced concepts such as risk-appropriate and science-based regulation, PBA offers a clearer pathway for operationalizing these approaches. It provides a structured yet flexible framework that bridges the gap between theoretical regulatory ideals and their practical implementation in the governance of emerging biotechnologies.

This article critically examines both the PP’s theoretical and practical implications and evaluates the PBA’s growing relevance. The analysis reveals that strict adherence to the PP, particularly in process-based regulatory systems, hindered innovation, delaying product approvals, and obstructing international regulatory harmonization. In contrast, the PBA offers a more adaptive, transparent, and proportionate framework, one that can evolve in tandem with scientific and technological progress.^97–98^

To strengthen GEd technology governance, this article proposes the following recommendations:

- Adopt a hybrid regulatory model that combines the risk-awareness of the PP with the flexibility and adaptability of the PBA. This balance ensures safety while enabling timely and proportionate regulatory responses.
- Clarify and update legal definitions in national legislation to distinguish GEd organisms and GMOs based on SDN terminologies. Such legal clarity will support science-based, product-focused regulation and reduce ambiguity in regulatory interpretation.
- Outline guiding principles for regulating GEd based on regions such as ASEAN, APEC, and European Union to avoid fragmentations. Product evaluations should include targeted science-based assessments of possible toxicity, allergenicity, off-target edits related to the characteristics of the final product. These assessments should be implemented through a tiered risk assessment framework, supported by inclusive stakeholder engagement to ensure informed decision-making.

Although research on the PBA remains limited, it presents a promising avenue for further study and practical exploration. For countries like Malaysia, South Korea, Indonesia and Taiwan, where regulatory reforms on GEd are currently under consideration, this presents a timely opportunity to adopt a forward-looking, principle-based regulatory framework.
